# Development of high-resolution melting curve analysis in rapid detection of *vanA* gene, *Enterococcus faecalis*, and *Enterococcus faecium* from clinical isolates

**DOI:** 10.1186/s41182-020-00197-9

**Published:** 2020-02-18

**Authors:** Sanaz Dehbashi, Hamed Tahmasebi, Parinaz Sedighi, Faeze Davarian, Mohammad Reza Arabestani

**Affiliations:** 1 Department of Microbiology, School of Medicine, Hamadan University of Medical Sciences, Pajoohesh junction, Hamadan, Iran; 2 Department of Microbiology, School of Medicine, Zahedan University of Medical Sciences, Zahedan, Iran; 3 Hamadan University of Medical Sciences, Hamadan, Iran; 4 School of Paramedical, Hamadan University of Medical Sciences, Hamadan, Iran; 5 Nutritious Research Center, School of Medicine, Hamadan University of Medical Sciences, Hamadan, Iran

**Keywords:** High-resolution melting curve analysis (HRMA), *Enterococcus faecium*, *Enterococcus faecalis*

## Abstract

**Background:**

High-resolution melting analysis (HRMA) is a novel molecular technique based on the real-time PCR that can be used to detect vancomycin resistance Enterococcus (VRE). The purpose of this study was to identify VRE species with HRMA in clinical isolates.

**Results:**

Out of 49 Enterococcus isolates, 11 (22.44%) *E. faecium* isolates and 19 (38.77%) *E. faecalis* isolates were detected. Average melting temperatures for *divIVA* in *E.faecalis*, *alanine racemase* in *E.faecium*, and *vanA* in VRE strains were obtained as 79.9 ± 0.5 °C, 85.4 ± 0.5 °C, and 82.99 ± 0.5 °C, respectively. Furthermore, the data showed that the HRMA method was sensitive to detect 10^0^ CFU/ml for the *divIVA*, *alanine racemase*, and *vanA* genes. Also, out of 49 Enterococcus spp., which were isolated by HRMA assay, 8 isolates (16.32%) of *E. faecium* and 18 isolates (36.73%) of *E. faecalis* were detected. The *vanA* gene was reported in 2 isolates (25%) of *E. faecium* and 9 isolates (50%) of *E. faecalis*.

**Conclusions:**

This study demonstrated that using the HRMA method, we can detect *E. faecium*, *E. faecalis*, and the *vanA* gene with high sensitivity and specificity.

## Background

High-resolution melting analysis (HRMA) is an innovative technology that has the potential to distinguish bacterial types (e.g., resistance types) based on variations in DNA methylation patterns. The HRMA is a simpler and more cost-effective way to characterize multiple bacterial samples and antibiotic resistance, comparing to other conventional methods such as culture and biochemical identification [1]. HRMA relies on a unique, highly saturated double-stranded DNA (dsDNA) binding dyes (e.g., EvaGreen) that can detect the presence of heteroduplexes which are formed during PCR [2]. Moreover, this technique measures the changes in the fluorescence intensity during melting with higher resolution than conventional melting analysis [3]. Consequently, minor variations in the sequence of PCR amplicons cause slight changes in the melt curves and are detected using a dedicated gene-scanning software [4]. During the HRMA method, this process is monitored using intercalating dyes [3]. The HRMA method is able to precisely and rapidly detect the DNA of pathogens. The accuracy and efficiency are the advantages of this method. The major challenge to develop this method is applying the most specific melt temperature for each target [5].

Vancomycin resistance Enterococcus (VRE) is an important cause of nosocomial infections [6]. *Enterococcus faecalis* and *Enterococcus faecium* were identified as the two most prevalent Enterococcus spp. causing healthcare-associated infections (HAI) [4, 6]. *E. faecium* has been reported as the most common Enterococcus spp. [6]. There are six main VRE phenotypes: *vanA*, *vanB*, *vanC*, *vanD*, *vanE*, and *vanG*, with a high prevalence reported for *vanA* and *vanB*. The *vanA* is the most common phenotype of VRE and demonstrates higher level of resistance to vancomycin and teicoplanin. The spread of the *vanA* gene from Enterococcus to *Staphylococcus aureus* in the same patient demonstrates the mobility of these resistance genes [4, 7]. Moreover, *divIVA* gene encodes a multifunctional protein, which plays an important role in cell division, biofilm formation, resistance, and viability in *E. faecalis.* The *divIVA* gene has a conserved N-terminal domain in *E. faecalis* which can be used as a detection target [8, 9].

Given the importance of different Enterococcus spp. in human, accurate and rapid identification of these species is important. Phenotypic tests, in addition to their time-consuming procedures and high costs, may be associated with some errors [10, 11]. Hence, the use of molecular techniques is essential more than ever. Although many experimental studies have been performed on different species of Enterococcus by the HRMA method, limited research has been done on VRE strains [4].

The purpose of this study was to evaluate HRMA as a rapid and accurate method for the identification of *E. faecium* and *E. faecalis*. Further, by optimizing the HRMA method, the *vanA* gene was identified in these species.

## Materials and methods

### Isolation and identification of *Enterococcus* species

Forty-nine (49) human clinical Enterococcus spp*.* isolated in this experimental-analytical study and collected from 374 clinical specimen including blood, sputum, urine, ulcers, and secretions from therapeutic centers of Hamadan University of Medical Sciences (Beheshti Hospital, Farshchian Hospital, and Be’sat Hospital) during April 2015 to August 2016. The samples were cultured on sheep blood agar (Merck, Germany) and incubated at 37 °C overnight. Then, the small, smooth, cream colonies were subcultured to bile esculin azide agar (Liofilchem, Italy), and also, biochemical tests including leucyl aminopeptidase (LAP), pyrrolidonyl arylamidase (PYR), and growth in 6.5% NaCl broth were performed. All clinical Enterococci were further identified to the species using conventional biochemical tests devised by Mahon, Textbook of Diagnostic Microbiology [12].

### Preparation of DNA extraction

To extract the DNA, a boiling method was used according to Tongeren et al. study [13]. Firstly, several colonies of Enterococcus spp. were suspended in 400 μl of deionized sterile water (Sigma, St. Louis, USA), vortexed for 10 s, and centrifuged for 5 min at 8000 rpm. Then, supernatants were removed, and 100 μl of 50 mM NaOH (Sigma, St. Louis, USA) was added, vortexed, and heated at 95 °C for 15 min. After that, 50 μl of 20 mM Tris buffer (Sigma, St. Louis, USA) was added and centrifuged at 1200 rpm at 4 °C. Finally, 100 μl of supernatants as genomic DNA were collected and used for molecular tests. The quality of genomic DNA extracted from the Enterococcus spp. was analyzed on 1% (w/v) agarose (Sigma, St. Louis, USA) gel in 1X TAE buffer, and the DNA concentration and UV absorption were measured in the wavelength of 260 and 280 nm by a spectrophotometer Nanodrop (Hangzhou Allsheng Instruments Co., Ltd., China).

### Optimization of PCR and sanger sequencing

PCR was carried out using *divIVA* (for *E. faecalis*), *alanine racemase* (for *E. faecium*), and *vanA* (for VRE strains) primers listed in Arabestani et al. [11] (Table 1) and Okolie et al. studies [14] in a final volume of 25 μl, containing 12.5 μl of 2X master mix (Ampliqon, Denmark), 1 μl of forward primers (10 pmol), 1 μl of reverse primers (10 pmol), 1 μl of DNA sample, and 9.5 μl of deionized sterile water (Sigma, St. Louis, USA). The ready-to-use Amplicon 2X master mix contains 2.5 mM MgCl_2_. DNA amplification was performed (Eppendorf Thermocycler, Germany) with thermal cycling conditions consisting of an initial denaturation step at 94 °C for 5 min, followed by 30 amplification cycles that included denaturation at 94 °C for 1 min, annealing at 58 °C for 1 min, and extension at 72 °C for 10 min**.***E. faecalis* NCTC13779*, E. faecium* NCTC7174, and *E. faecium* ATCC51559 (for *vanA* gene) were used as positive controls. Forward and reverse primers for *divIVA*, *alanine racemase*, and *vanA* were used as sequencing primers using the Eppendorf Thermocycler (Germany). All PCR products were sent to Pishgam Company (Tehran, Iran) for sequencing.

**Table 1:** Oligonucleotide sequences used in this study

Gene	Target	Sequence of primers (5′ to 3′)	Melting (Tm)	Product size (bp)	Ref
*divIVA*	*E. faecalis*	F: ACGTGTCTTCCATCAACGCT R: ACTGCTGTATGTTTGTCTCCGA	79.9	123	[11]
*Alanine racemase*	*E. faecium*	F: ATCCCTCTGGGCACGCAC R: ACATACACGCCCAATCGTTTC	85.4	248	[11]
*vanA*	Vancomycin	F: GCTGTGAGGTCGGTTGTG R: GCTCGACTTCCTGATGAATACG	82.9	101	[11]

### Evaluation of sensitivity and specificity of real-time PCR

A two-step amplification PCR assay was performed on a Step One-Plus® 96 instrument (ABI Step One-Plus, USA) using the EvaGreen dye (Takara Biomedicals, Kyoto, Japan). The amplification starts with an initial denaturation at 95 °C for 15 min, followed by 40 cycles of 95 °C for 30 s and 57 °C for 30 s. One cycle of melt curve step was conducted by ramping the temperature from 60 °C to 90 °C. The melt curve plot was prepared by plotting the negative derivative of fluorescence (− Rn) versus temperature. To determine the sensitivity of each primer in the real-time PCR assay, the DNAs isolated from *E. faecalis* NCTC13779*, E. faecium* NCTC7174, and *E. faecium* ATCC51559 (for *vanA* gene) were serially diluted, starting from 10^7^ to 10^0^ CFU/ml (0.5 McFarland 1.5 × 10^8^ CFU/ml). Two microliters of each serially diluted DNAs were applied in triplicate with each primer in singleplex/multiplex for the real-time PCR. PCR efficiency was calculated from the slope of the standard curve using the formula *E* = 10^(−1/slope)^−1. The primer efficiency range between 90 and 110% was considered as reliable. In this study, the efficiency of each gene was calculated by the ABI® 96 Software 3.2.0 (ABI Step One-Plus, USA).

### Optimization of real-time PCR and HRMA

Normalized and difference graphs were generated to assess the ability of the HRMA method to differentiate among bacterial strains. HRMA assay amplification was performed, using a real-time PCR instrument (ABI Step One-Plus, USA). Reactions were prepared in a total volume of 20 μl that included 4 μl of 5X HRMA Master Mix (HOT FIREPol® EvaGreen HRMA Mix), 1 μl of each primer (20 pmol), 1 μl of bacterial DNA, and 13μl of DEPC-treated water (Sigma, St. Louis, USA). The amplification protocol involved a 10 min hold at 95 °C followed by 40 amplification cycles comprising of 95 °C denaturation for 15 s, annealing at 58 °C for 30 s, and extention at 72 °C for 10 s. The HRMA reactions were carried out in triplicate for each isolate tested. After amplification, high-resolution melting was carried out on the PCR amplicons which were generated. The first part of the melting process involved a brief denaturation of samples at 95 °C for 1 min and rapid annealing. Following this, the temperature was again slowly increased and HRMA curve data obtained from 60 °C to 95 °C.

### Data analysis

The correlation coefficients (*R*^2^) and slope values were obtained from the standard curve, and the efficiency (*E*) of PCR was calculated according to the equation *E* = 10^–1/slope^−1.

The ABI Step One-Plus software version 2.3 (Thermo Fisher Scientific, Inc., USA) generated the amplification and melt curves, cycle threshold (CT), products’ melt temperature (Tm), standard curve, and unknown sample quantification data. HRMA data analyzed by the ABI Step One-Plus High-Resolution Melt (HRMA) software V3.01 (ABI Thermo Fisher Scientific, Inc., USA).

## Results

Out of 49 Enterococcus spp., 11 (22.4%) *E. faecium* and 19 (38.8%) *E. faecalis* were isolated. Nineteen isolates (38.8%) were not detectable by phenotypic tests. Out of 11 isolates of *E. faecium*, 8 isolates (72.72%) were obtained from female patients, and 3 isolates (27.27%) were collected from male patients. Also, 2 isolates (27.27%) were collected from blood, 1 isolate (9.09%) was isolated from ulcer, 1 isolate (9.09%) was obtained from sputum, and 7 isolates (63.63%) were collected from urine. Among 19 *E. faecalis* isolates, 13 isolates were isolated from female patients and 6 isolates were obtained from male patients. Moreover, 7 isolates (36.84%) were obtained from blood, 2 isolates (10.05%) were isolated from ulcer, 2 isolates (10.52%) were collected from sputum, and 9 isolates (57.89%) were isolated from urine were collected.

### Species identification by PCR and sequencing

The results of sequencing were blasted and determined as *E. faecalis* NCTC13779 with NZ_UGJA01000003.1 accession number, *E. faecium* NCTC7174 with LR134337.1 accession number, and *E. faecium* ATCC51559 with accession number of JSVT00000000.

### The analytical sensitivity and specificity of the real-time PCR

**Fig. 1:**
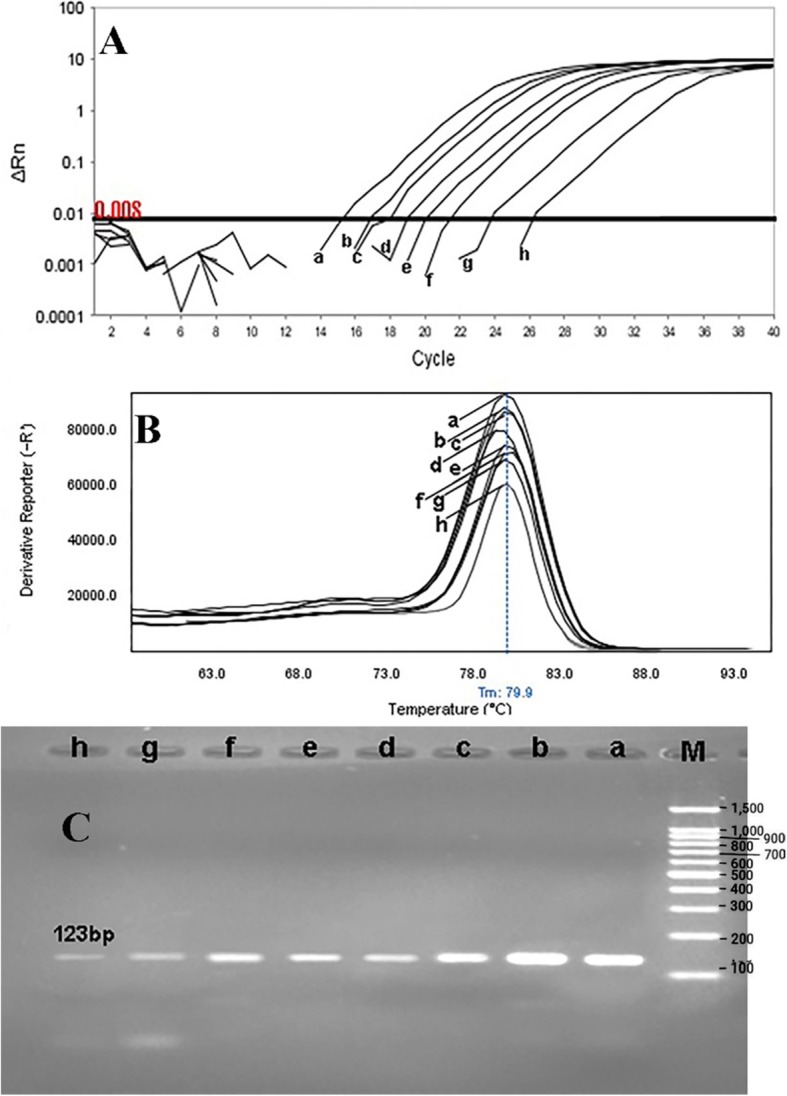
Sensitivity of real-time PCR for primers used to detect of *E. faecalis (divIVA* gene) with a melting point of 79.9 ± 0.5 °C and amplification curve. *E. faecalis NCTC13779* (as standard bacteria) with the concentration of 0.5 McFarland (1.5 × 10^8^ CFU/ml) was provided into serial dilutions of 10^7^ to 10^0^ CFU/ml. a 10^7^, b 10^6^, c 10^5^, d 10^4^, e 10^3^, f 10^2^, g 10^1^, and h 10^0^. **a** Amplification curve, **b** Melting curve profile, and **c** corresponding agarose gel (1.5%) electrophoresis for real-time PCR amplification of *divIVA* gene. Horizontal lines represent cycle threshold of real-time PCR. One peak with a shoulder corresponds to genomic DNA amplification; no peak corresponds to no amplification. SYBR Green I color and single-tube reaction were used in this test. Also, real-time PCR was performed as single step

**Fig. 2:**
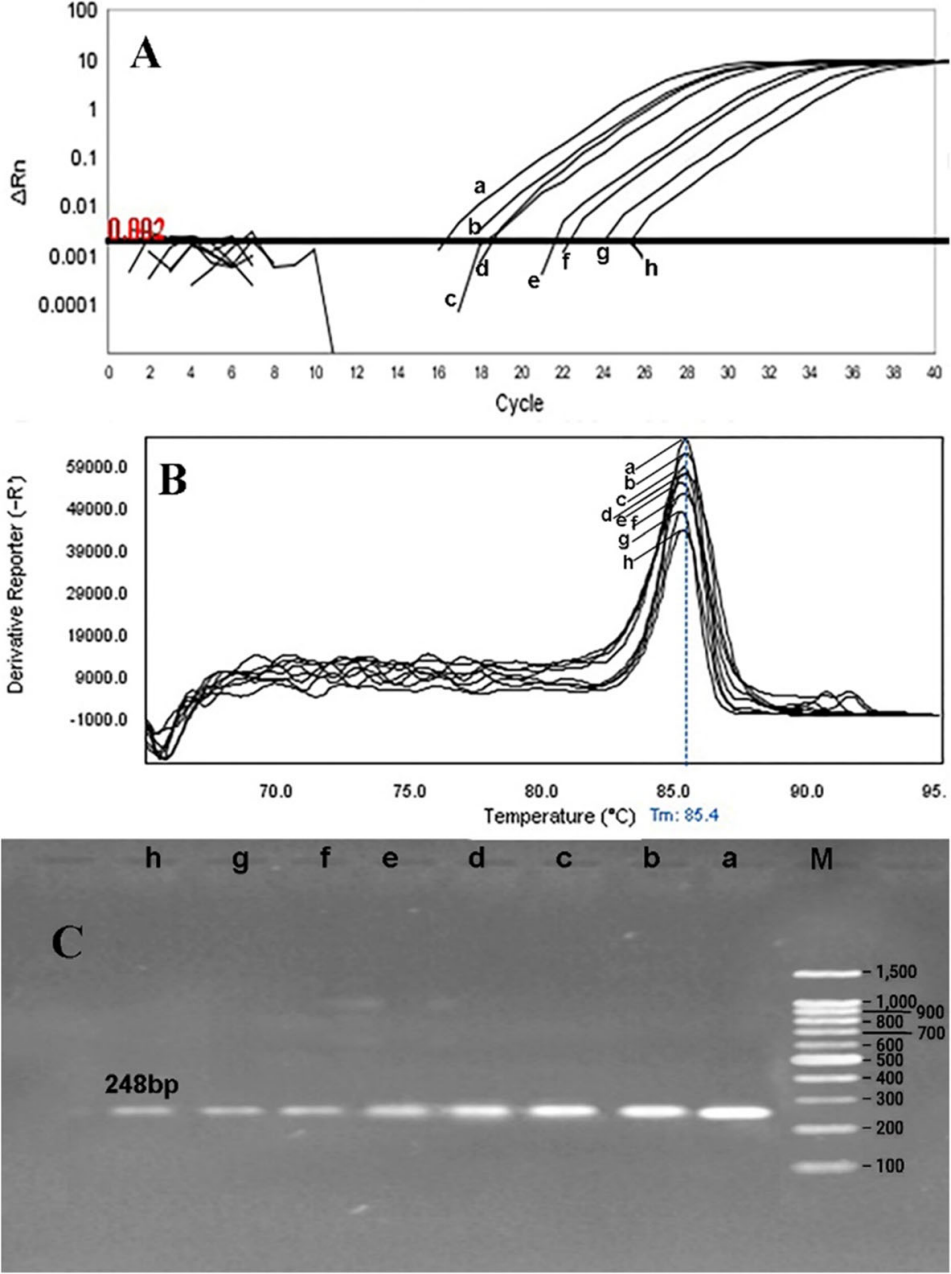
Sensitivity of real-time PCR for primers used to detect of *E. faecium* (*alanine racemase* gene) with a melting point of 85.4 ± 0.5 °C and amplification curve. *E. faecium NCTC7174* (as standard bacteria) with the concentration of 0.5 McFarland (1.5 × 10^8^ CFU/ml) was provided into serial dilutions of 10^7^ to 10^0^ CFU/ml. a 10^7^, b 10^6^, c 10^5^, d 10^4^, e 10^3^, f 10^2^, g 10^1^, and h 10^0^. **a** Amplification curve **b** Melting curve profile, and **c** corresponding agarose gel (1.5%) electrophoresis for real-time PCR amplification of *alanine racemase* gene. Horizontal lines represent cycle threshold of real-time PCR. One peak with a shoulder corresponds to genomic DNA amplification; no peak corresponds to no amplification. SYBR Green I color and single-tube reaction were used in this test. Also, real-time PCR was performed as single step

**Fig. 3:**
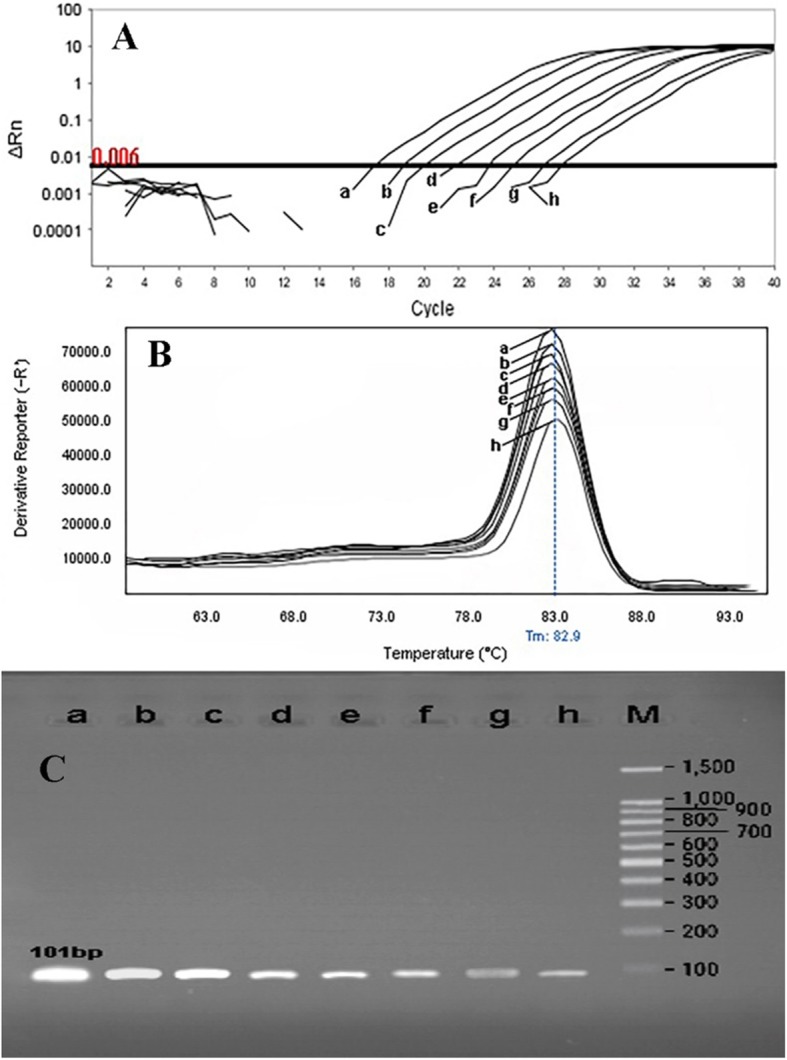
Sensitivity of real-time PCR for primers used to detect of VRE strains (*vanA* gene) with a melting point of 82.99 ± 0.5 °C and amplification curve. *E. faecium ATCC51559* (as standard bacteria) with the concentration of 0.5 McFarland (1.5 × 10^8^ CFU/ml) was provided into serial dilutions of 10^7^ to 10^0^ CFU/ml. a 10^7^, b 10^6^, c 10^5^, d 10^4^, e 10^3^, f 10^2^, g 10^1^, and h 10^0^. **a** Amplification curve, **b** Melting curve profile, and **c** corresponding agarose gel (1.5%) electrophoresis for real-time PCR amplification of *vanA* gene. Horizontal lines represent cycle threshold of real-time PCR. One peak with a shoulder corresponds to genomic DNA amplification; no peak corresponds to no amplification. SYBR Green I color and single-tube reaction were used in this test. Also, real-time PCR was performed as single step

**Fig. 4:**
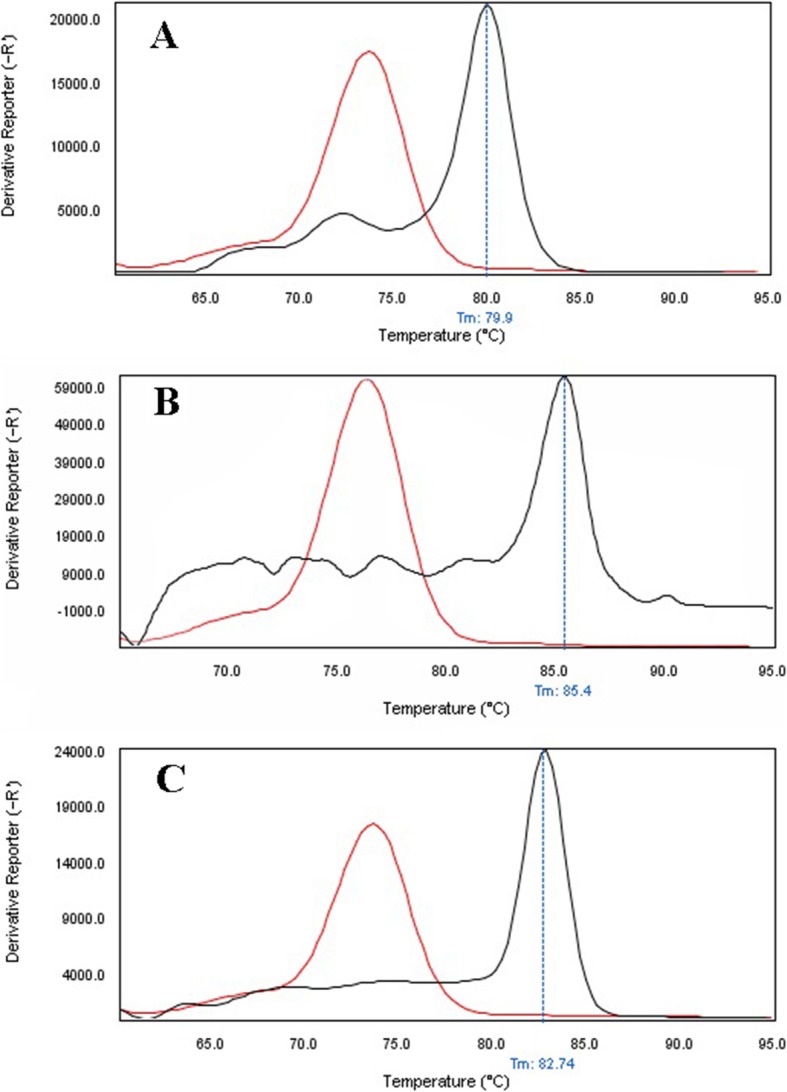
Specificity of real-time PCR for primers used to detect of *E. faecalis*, *E. faecium*, and VRE stains. Melting curve analysis showing the melting temperature peaks (Tm) of *E. faecalis* (**a**), *E. faecium* (**b**), and VRE stains (**c**). **a** blacks’ lane, *E. faecalis NCTC13779*; red lane, *Streptococcus pneumoniae ATCC49619*. **b** blacks’ lane, *E. faecium NCTC7174*; red lane, *Streptococcus pneumoniae ATCC49619*. **c** blacks’ lane, VRE strains; red lane, *Streptococcus pneumoniae ATCC49619*

The primer efficiency was obtained as following: *divIVA* efficiency = 99.89% for *E. faecalis* NCTC13779, *alanine racemase* efficiency = 100.01% for *E. faecium* NCTC7174, and *vanA* efficiency = 101.09% for *E. faecium* ATCC51559. The sensitivity of the real-time PCR assay was found to be 10^7^ to 10^0^ CFU/ml for all standard strains (0.5 McFarland 1.5 × 10^8^ CFU/ml) (Fig. 1). In all dilutions (in standard strains), melt curve assay showed three distinctive and well separated peaks. These separated peaks of *divIVA* gene for *E. faecalis* (Fig. 2), *alanine racemase* gene for *E. faecium* (Fig. 3), and *vanA* gene for VREs (Fig. 4) were formed with the average melting temperatures of amplicons of 79.9 ± 0.5 °C, 85.4 ± 0.5 °C, and 82.99 ± 0.5 °C, respectively. All of these melting temperatures were completely consistent with the results of primer blasts in NCBI database. The lengths of the *vanA*, *alanine racemase*, and *divIVA* primers were 101 bp, 248 bp, and 123 bp, respectively. And as shown in Figs. 3 and 4, *vanA* primer had the highest sensitivity and specificity.

### Detecting isolates by HRMA assay

**Fig. 5:**
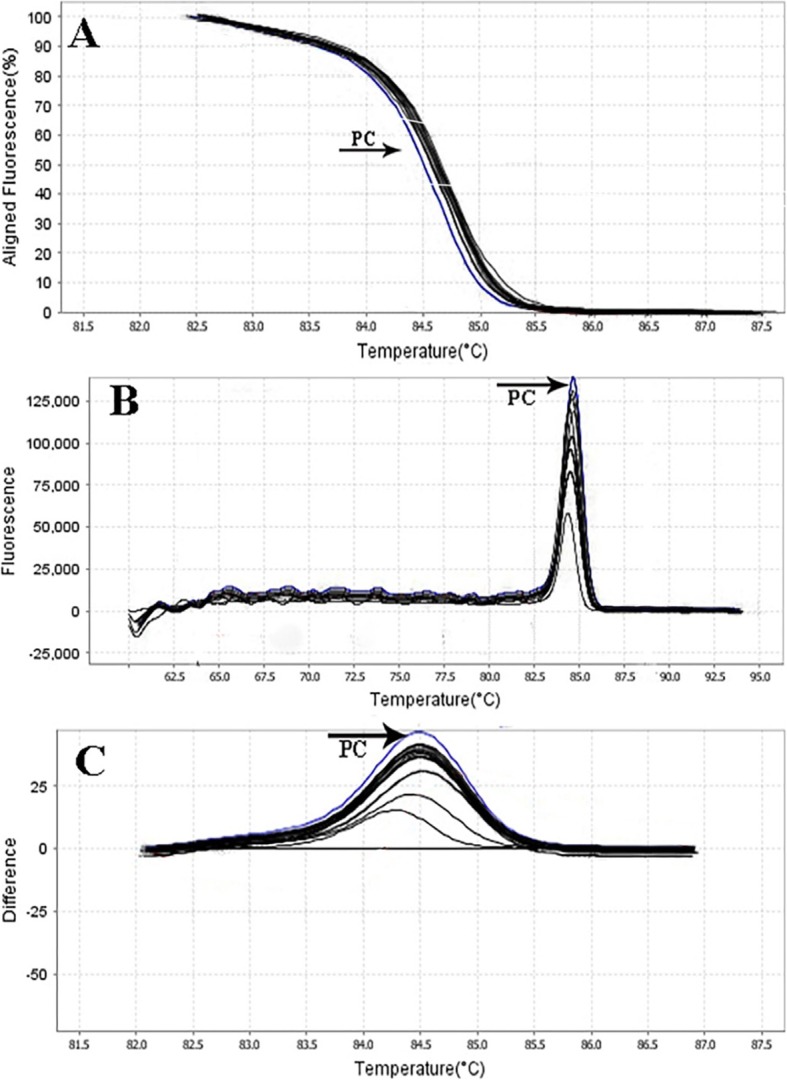
HRMA graphs corresponding to one high-resolution melting analysis of a subset of clinical specimens (*n* = 8). Curves of tested samples previously identified as *E. faecium* are shown in black lanes. DNA samples in this study were prepared and amplified successfully using the EvaGreen dye-based method in the ABI instrument. Primer-specific melting peaks (Tm) were obtained via HRM analysis, allowing the differentiation of all investigated β-lactamase enzymes. Due to the highly saturating EvaGreen dye and the HRMA analysis, the accuracy of the resolution was ± 0. 1–0.5 °C

**Fig. 6:**
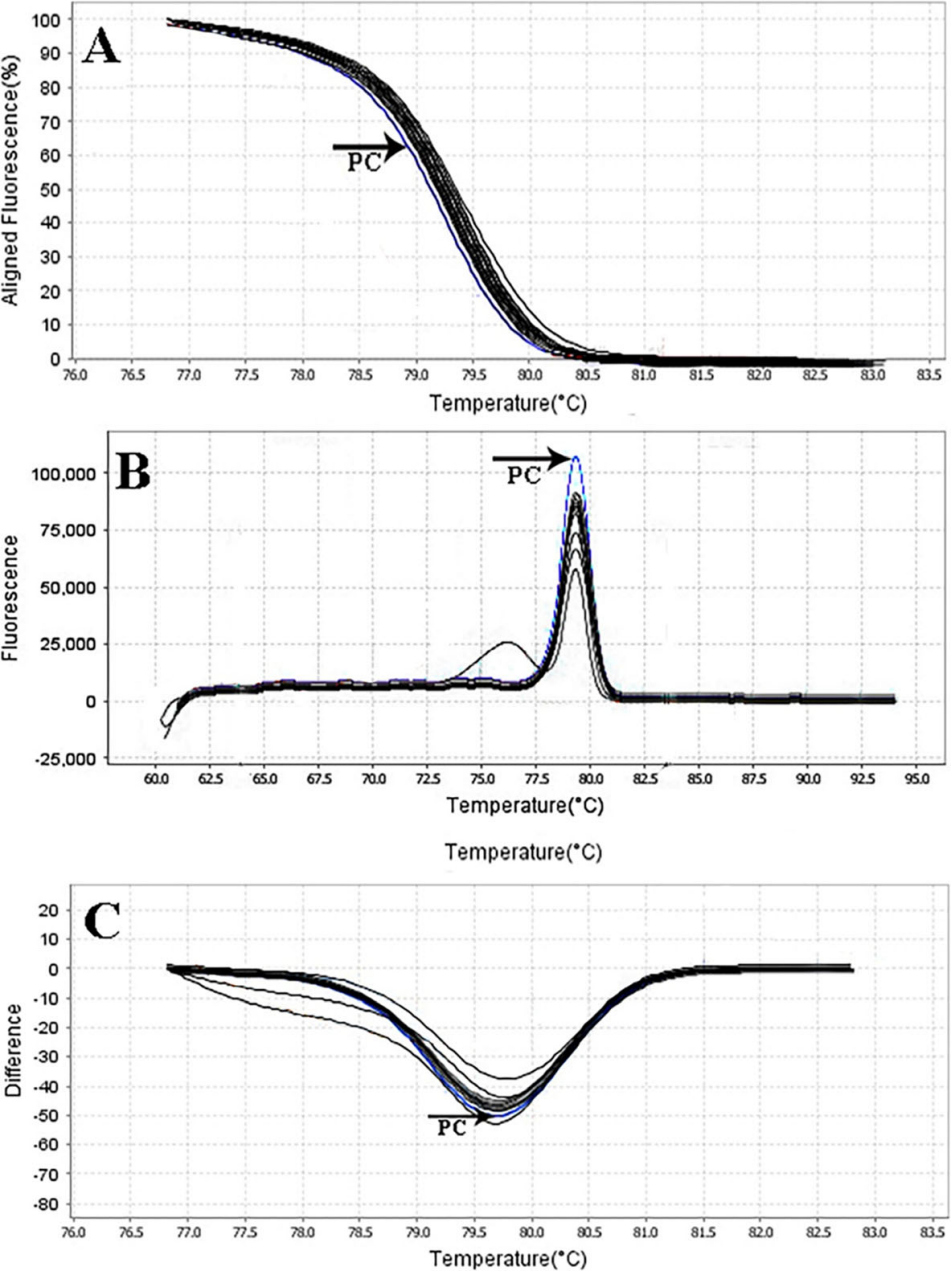
HRMA graphs corresponding to one high-resolution melting analysis of a subset of clinical specimens (*n* = 18). Curves of tested samples previously identified as *E. faecalis* are shown in black lanes. DNA samples in this study were prepared and amplified successfully using the EvaGreen dye-based method in the ABI instrument. Primer-specific melting peaks (Tm) were obtained via HRM analysis, allowing the differentiation of all investigated β-lactamase enzymes. Due to the highly saturating EvaGreen dye and the HRMA analysis, the accuracy of the resolution was ± 0. 1–0.5 °C

**Fig. 7:**
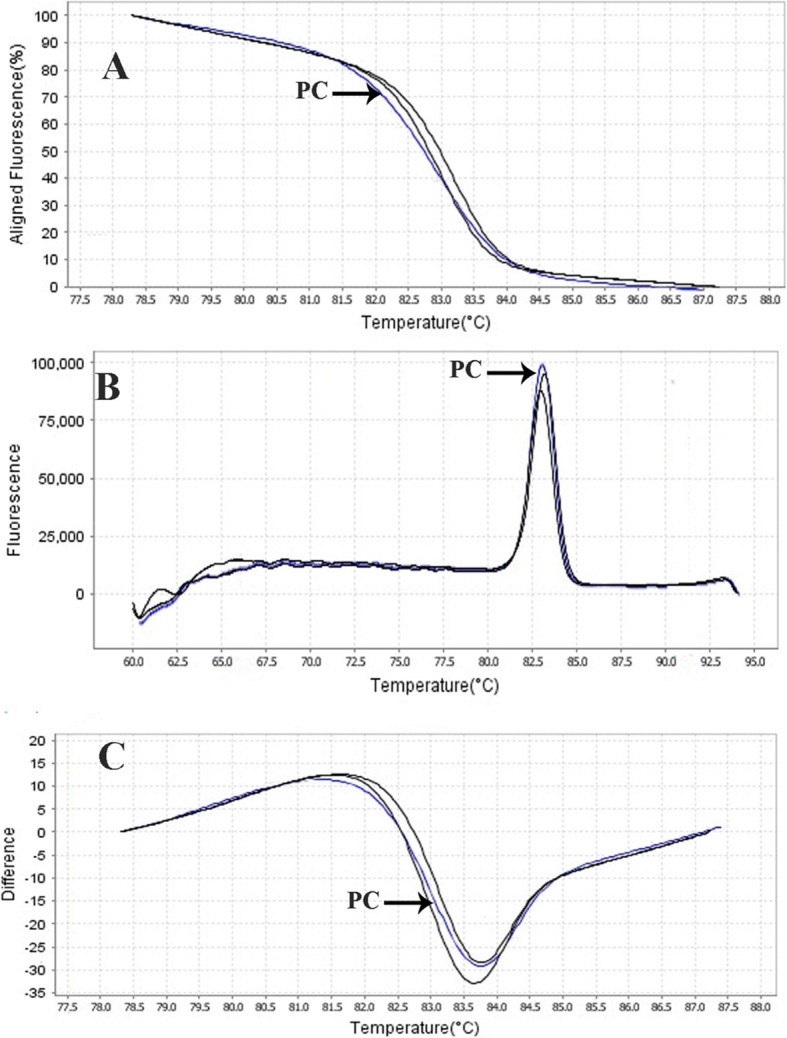
HRMA graphs corresponding to one high-resolution melting analysis of a subset of clinical specimens of *E. faecium*. DNA samples in this study were prepared and amplified successfully using the EvaGreen dye-based method in the ABI instrument. Primer-specific melting peaks (Tm) were obtained via HRM analysis, allowing the differentiation of all investigated β-lactamase enzymes. Due to the highly saturating EvaGreen dye and the HRMA analysis, the accuracy of the resolution was ± 0. 1–0.5 °C

**Fig. 8:**
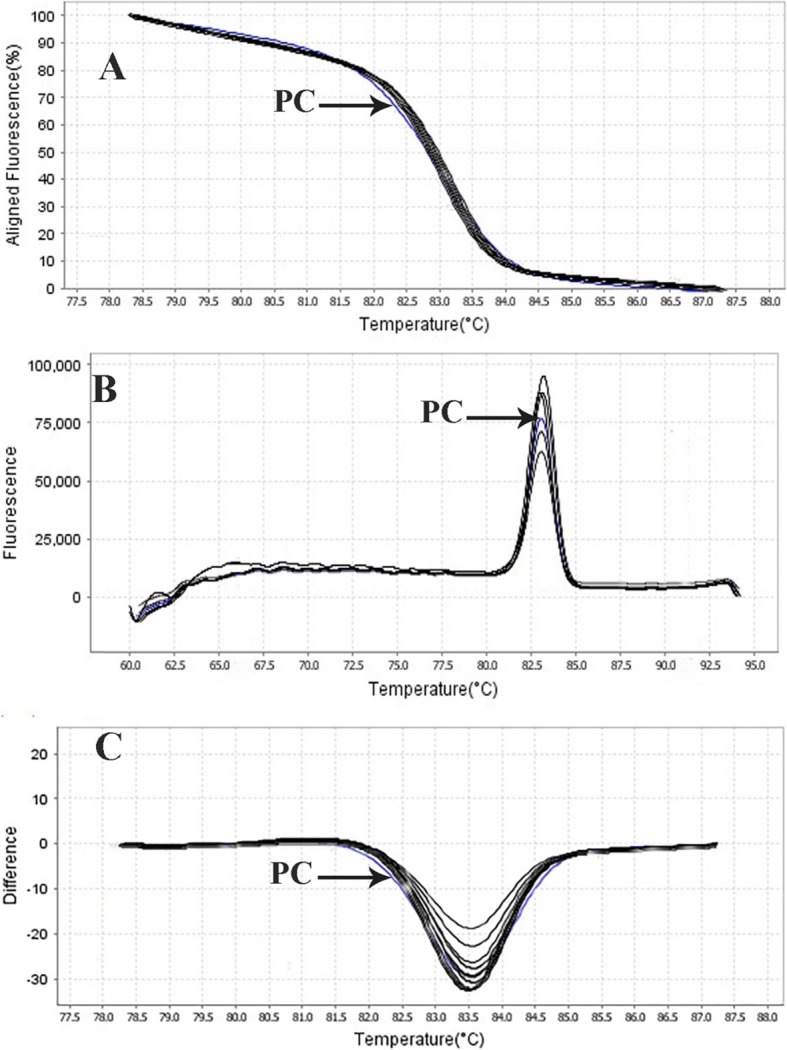
HRMA graphs corresponding to one high-resolution melting analysis of a subset of clinical specimens of *E. faecalis*. DNA samples in this study were prepared and amplified successfully using the EvaGreen dye-based method in the ABI instrument. Primer-specific melting peaks (Tm) were obtained via HRM analysis, allowing the differentiation of all investigated β-lactamase enzymes. Due to the highly saturating EvaGreen dye and the HRMA analysis, the accuracy of the resolution was ± 0. 1–0.5 °C

The results showed that out of 11 clinical isolates (22.5%) of *E. faecium* identified by the phenotypic method, HRMA test was positive in 8 isolates (16.32%) (Fig. 5). Moreover, out of the 19 clinical isolates (38.77%) of *E. faecalis*, HRMA test was positive in 18 isolates (36.73%) (Fig. 6). In addition, based on the HRMA results, *vanA* gene was positive in 2 isolates (25%) of *E. faecium* (Fig. 7) and 9 isolates (50%) of *E. faecalis* (Fig. 8). Out of 8 isolates of *E. faecalis*, 2 isolates (25%) obtained from blood and 6 isolates (75%) collected from urine were detected by the HRMA method. The HRMA-detected isolates of *E. faecium* were obtained from blood (7 isolates, 28.88%), ulcer (1 isolate, 5.55%), sputum (2 isolates, 55.55%), and urine (8 isolates, 55.55%).

## Discussion

Rapid and cost-effective detection of *E. faecium*, *E. faecalis*, and VRE strains is important for the health care settings [6]. The gold standard for the identification of Enterococcus spp. is biochemical identification. This traditional method of identification is time-consuming and labor-intensive. Also, it takes up to 5 days or more [4]. On the other hand, HRMA real-time PCR has the capacity to detect more than one target in a single reaction, thus it saves time and cost. As it is shown in Figs. 1, 2 and 3, the DNA templates were amplified well. The increase of the fluorescence signals was observed at the 14 to 28 cycles, and the plateau was reached for all samples. Nevertheless, the primers (designed in previous study [11]) have been validated to show 99.89% specificity for *E. faecalis* and 100.01% for *E. faecium* in clinical isolates of Enterococci. Furthermore, based on Figs. 1, 2, and 3 for sensitivity and Fig. 4 for specificity, our analysis suggested that primers which were used to amplify an amplicon with 50 to 300 bp in length and target sites for primer design play an important role in enhancing the sensitivity and specificity of detection by HRMA. Studies by Ramirez et al. [8] and Priyadarshi et al. [15] showed that target sites such as *divIVA* and *alanine racemase* have the best sensitivity and specificity to identify *E. faecalis* and *E. faecium* species, respectively. The potential of specific melting domains provides an additional advantage of using small amplicons with HRM analysis, although it is not guaranteed to occur in every case, depending on the exact nucleotide composition and the sequence of the amplicon.

In Figs. 5, 6, 7, and 8, the melting profile derivative plot was shown. Nonetheless, these results demonstrated that the melting temperature for *divIVA* primer was 79.9 ± 0.5 °C and for *alanine racemase* primer was 85.4 ± 0.5 °C, and HRMA assay detected 10^0^ CFU/ml of *E. faecalis* and *E. faecium*. Ozbak [4] reported that the HRMA method for the detection of Enterococcus spp. in standard strains showed a sensitivity of 100%, which was much higher than the sensitivity achieved by biochemical methods. Various studies in Spain [16], Sweden [17], and Finland [3] determine that for identification of different Enterococcus spp., HRMA method is more sensitive than culture method and can efficiently detect VRE strains in standard strains.

The optimal concentration of MgCl_2_ for the real-time PCR was determined as 2.5 mM. A proper MgCl_2_ concentration in a PCR reaction is one of the most important factors in building a distinguishable plot for determining Enterococcus species [18, 19]. On the other hand, the type of Master Mix and Dye also play an important role in the results and sensitivity of HRMA. In this study, EvaGreen was used as dye; Khan et al. [20] and Eischeid [2]. confirmed that this dye is more sensitive than the SYBRGreen. They concluded that the type of fluorescent Dye plays a significant role in the development of the sensitivity and specificity of the HRMA method. They also modified the primer sequences to reduce the overall size of the amplified product as HRMA is thought to be more consistent when the amplified product is < 500 bps. However, the results of the present study showed that primer length is also important in the detection of different bacteria using the HRMA method (Table 1).

Rapid and accurate detection of VRE is critical to block the transmission of the infection. Moreover, the emergence of vancomycin-resistant *Staphylococcus aureus*, which has acquired the *vanA* gene, emphasizes the importance of VRE infection control. The detection of VRE strains by HRMA is the most specific test and is useful for the detection of VRE with low tolerance. Therefore, the present paper showed that the *vanA* gene was positive in 2 isolates (25%) of *E. faecium* and 9 isolates (50%) of *E. faecalis* by HRMA technique, also, primer specificity for *vanA* gene was 100.01% and melting temperature for this gene observed at 79.9 ± 0.5 °C. Lata et al. [21] reported a real-time PCR assay for detection of VRE with a sensitivity of 10^3^ genomic equivalents. Cha et al. [22] reported a multiplex real-time PCR assay for detection of viable VRE with sensitivity of 10^2^ CFU/ml. Young et al. [23] and Babady et al. [7] reported a lower detection limit of 10^4^ CFU/ml and 10^2^ CFU/ml, respectively. In the present study, a detection limit of 10^1^ and 10^0^ CFU/ml equivalents was achieved in singleplex and multiplex assay format, respectively. The detection limit of the real-time PCR assay developed in this study for detection of VRE comparable to results obtained by Ozbak [4] and Deshpande et al. [24].

Identification of different Enterococcus species and antibiotic resistance by phenotypic method has several disadvantages. To explain, the conventional susceptibility methods require at least 24 h following an overnight inoculation (16–20 h) and in the case of agar-based methods (disk diffusion, *E* test), poor diffusion of vancomycin has been observed and caused false negative (indicating susceptibility rather than resistance) results. In one study, Váradi et al. [25] found that phenotypic methods for the identification of pathogenic bacteria are associated with limitations such as error in detection, low sensitivity and specificity, and low speed in identifying large numbers of samples. Moreover, the major challenge for all methods is detecting heteroresistant species. Heteroresistant is defined as a subpopulation of bacteria in a phenotypically susceptible population and is associated with vancomycin resistance. Based on Chen et al. [26] study, HRMA has been shown to provide a successful platform for the identification of microbial pathogens and to discriminate heteroresistant strains that confer a decrease in susceptibility to antimicrobials. HRMA has the potential to be a powerful tool in the clinical microbiology laboratory, providing rapid detection of genetic determinants conferring antibiotic resistance to complement current phenotypic antimicrobial susceptibility testing methods. Besides, HRMA is a simpler and more cost-effective way to characterize multiple samples [27].

The early detection of VRE pathogens has a great impact on the clinical outcome of the infection. We have improved the identification of Enterococcus spp. by means of the HRMA investigation, thereby achieving the distinction of 99% of Enterococcus pathogens in sepsis. Thus, the protocol confer the possibility for the rapid detection and reliable differentiation of the *E. faecium* and *E. faecalis*, species most frequently isolated from clinical samples in urine infections. However, in the present study, laboratory conditions had a direct effect on the results. DNA melting temperature and primer concentration were also determined according to the ability of the real-time PCR to detect different bacteria. Therefore, the HRMA method should be optimized in vitro for the detection of VRE strains and different strains of Enterococcus.

### Study limitations

There are two general limitations in our research that should be considered in subsequent studies: (a) limited dilutions were used to determine the sensitivity and (b) using a two-step reaction instead of a one-step reaction can affect the results. Hence, using more dilutions and low concentration of bacterial suspensions could give better results. Although the use of HRMA technique in some cases is at high cost, use of the real-time multiplex PCR method can play an important role to reduce the costs. Therefore, it is suggested that in future studies, the effectiveness of HRMA method in comparison with phenotypic methods for the diagnosis of other bacteria involved in hospital infections should be considered.

## Conclusions

Real-time PCR technique as an alternative test has been widely employed in detections of Enterococcus spp. during recent decades, as it offers accurate, rapid, and low-cost detection. The developed HRMA assay is easy operating and requires no use of probe, which makes it a useful tool for epidemiology analysis and laboratory use. Overall, HRMA-based PCR methods worked effectively in detecting Enterococcus spp. in various clinical samples. Hence, this study provided rapid detection methods for the patient to detect VRE strains in clinical isolates. Owing to the simplicity and ease of use, HRMA application in the areas of clinical diagnostics has also increased. This assay can be further tested with a large amount of pure or mixed strains and contaminated clinical samples.

## Data Availability

The data can be accessible to the interested researchers by the corresponding authors on reasonable request.

## Abbreviations

*E. faecalis*: *Enterococcus faecalis*
*E. faecium*: *Enterococcus faecium*
HRMA: High-resolution melting analysis
VRE: Vancomycin resistance Enterococcus
